# Gut Microbiota and Short-Chain Fatty Acids in Cardiometabolic HFpEF: Mechanistic Pathways and Nutritional Therapeutic Perspectives

**DOI:** 10.3390/nu18020321

**Published:** 2026-01-20

**Authors:** Antonio Vacca, Gabriele Brosolo, Stefano Marcante, Sabrina Della Mora, Luca Bulfone, Andrea Da Porto, Claudio Pagano, Cristiana Catena, Leonardo A. Sechi

**Affiliations:** 1 Clinica Medica, Department of Medicine, University of Udine, 33100 Udine, Italy; antonio.vacca94@gmail.com (A.V.); gabriele.brosolo@uniud.it (G.B.); stefanomarcante@outlook.it (S.M.); dellamora.sabrina@spes.uniud.it (S.D.M.); luca.bulfone1@gmail.com (L.B.); andrea.daporto@uniud.it (A.D.P.); claudio.pagano@uniud.it (C.P.); cristiana.catena@uniud.it (C.C.); 2 European Excellence Center for Arterial Hypertension, Department of Medicine, University of Udine, 33100 Udine, Italy; 3 Translational Approaches in Heart Failure and Cardiometabolic Disease, Max Delbrück Center for Molecular Medicine in the Helmholtz Association (MDC), 13125 Berlin, Germany; 4 Diabetes and Metabolism Unit, Department of Internal Medicine, Health Integrated Agency of Friuli Centrale, 33100 Udine, Italy

**Keywords:** heart failure with preserved ejection fraction, gut microbiota, short-chain fatty acids, gut–heart axis, cardiometabolic disease, inflammation, dietary fibers, microbiota-targeted therapy

## Abstract

Heart failure with preserved ejection fraction (HFpEF) accounts for more than half of the cases of HF worldwide. Among the different phenotypes, cardiometabolic HFpEF has the highest prevalence. Cumulative insults related to cardiometabolic comorbidities—obesity, hypertension and type 2 diabetes—create a milieu of metabolic derangements, low-grade systemic inflammation (i.e., metainflammation), endothelial dysfunction, and coronary microvascular disease. Emerging data indicate that the gut–heart axis is a potential amplifier of this process. Cardiometabolic comorbidities promote gut dysbiosis, loss of short-chain fatty acid (SCFA)-producing taxa, and disruption of the intestinal barrier, leading to endotoxemia and upregulation of pro-inflammatory pathways such as TLR4- and NLRP3-mediated signaling. Concomitantly, beneficial gut-derived metabolites (acetate, propionate, butyrate) decrease, while detrimental metabolites increase (e.g., TMAO), potentially fostering myocardial fibrosis, diastolic dysfunction, and adverse remodeling. SCFAs—acetate, propionate, and butyrate—may exert pleiotropic actions that directly target HFpEF pathophysiology: they may provide a CPT1-independent energy substrate to the failing myocardium, may improve lipid and glucose homeostasis via G protein-coupled receptors and AMPK activation, and may contribute to lower blood pressure and sympathetic tone, reinforce gut barrier integrity, and act as anti-inflammatory and epigenetic modulators through the inhibition of NF-κB, NLRP3, and histone deacetylases. This review summarizes current evidence linking gut microbiota dysfunction to cardiometabolic HFpEF, elucidates the mechanistic role of SCFAs, and discusses nutritional approaches aimed at enhancing their production and activity. Targeting gut–heart axis and SCFAs pathways may represent a biologically plausible and low-risk approach that could help attenuate inflammation and metabolic dysfunctions in patients with cardiometabolic HFpEF, offering novel potential therapeutic targets for their management.

## 1. Introduction

Heart failure (HF) represents a rapidly expanding global healthcare challenge associated with high morbidity and mortality rates. Considered as a clinical syndrome of symptoms and signs of cardiac origin rather than a single pathological entity, it affects 64 million people worldwide [1]. More than half of these patients meet HF with preserved ejection fraction (HFpEF) criteria (clinical HF with LVEF ≥ 50%), with the highest rates being detected in industrialized countries [1,2]. HFpEF incidence rises sharply with increasing age, affecting around 1–1.5% of the general population and nearly 1 in 10 individuals over 45 years old [1]. Most importantly, its increasing prevalence is related to the ongoing epidemics of cardiometabolic risk factors, such as obesity, hypertension, and type 2 diabetes (T2D) [3]. Indeed, the risk of developing HFpEF has a stronger association with cardiometabolic disorders than HFrEF [4]. Other important comorbidities associated with HFpEF are atrial fibrillation and chronic kidney disease [3], which further contribute to the overall burden of comorbidities that characterize the pathophysiology of HFpEF.

The complex interplay between comorbidities, increasing age, and environmental factors generates different clinical phenotypes of HFpEF, the cardiometabolic/obese HFpEF being the most prevalent form [4,5]. These cumulative insults induce metabolic derangements (insulin resistance, lipotoxicity), oxidative stress, and a chronic and low-grade systemic inflammation (i.e., metainflammation) that is pivotal in affecting myocardial structure and function [2,6]. A comprehensive model [7] indicates that increased pro-inflammatory cytokines promote the differentiation of fibroblasts into collagen-secreting myofibroblasts and enhance extracellular matrix degradation, leading to increased myocardial stiffness and coronary microvascular dysfunction (CMD) [7]. Endothelial inflammation in small coronary vessels reduces nitric oxide (NO) and cyclic guanosine monophosphate (cGMP) bioavailability, leading to the hypophosphorylation of the giant sarcomeric protein titin, which further increases myocardial stiffness and impairs diastolic function [7]. Resulting key features include myocardial energy impairment (e.g., mitochondrial dysfunction), increased fibrosis, extracellular matrix deposition, higher myocyte resting tension, and arterial stiffening, all contributing to elevated filling pressures despite preserved ejection fraction [8,9,10,11].

Emerging evidence points to the gut–heart axis as a key contributor in the pathophysiology of HFpEF. The great burden of comorbidities lays the groundwork for deep changes in the composition of gut microbial community and its metabolites (i.e., dysbiosis). Gut dysbiosis increases intestinal permeability, promotes endotoxemia, and alters microbial metabolite production, which may contribute to systemic inflammation and fibrosis central to HFpEF pathophysiology [12,13]. Therefore, recent attention has turned to gut microbiota-derived metabolites as potential modulators of systemic inflammation and overall cardiometabolic health. Among them, short-chain fatty acids (SCFAs)—principally acetate, propionate, and butyrate—have emerged as key signaling molecules. SCFAs exert pleiotropic actions, including improvements in cardiac metabolism, lipids and glucose homeostasis, modulation of blood pressure (BP), and attenuation of inflammation. Such mechanisms are particularly beneficial in cardiometabolic HFpEF, driven by metainflammation and metabolic dysregulation. Gut dysbiosis and alterations in SCFAs emerge as biologically plausible amplifiers of cardiometabolic HFpEF, suggesting the therapeutic potential of restoring SCFA homeostasis. This review provides an overview of the current evidence on gut microbiota dysfunction in cardiometabolic HFpEF, synthesizing mechanistic insights and translational relevance of SCFAs restoration, with a focus on their metabolic and non-metabolic pathways underlying their potential translational relevance.

## 2. Gut Microbiota: Composition and Functions

The human gut hosts trillions of mutualistic microorganisms, collectively referred to as the gut microbiota, which has been proposed to function as a virtual organ sustaining host physiology [14,15]. The gut microbiota encompasses diverse microorganisms from the domains *Bacteria*, *Archaea*, and *Eukarya*. Of these, *Bacteria* are the most numerous and are arranged into functional communities, with phyla of *Firmicutes* and *Bacteroides* making up the majority (>90%), followed by smaller populations of other phyla like *Actinobacteria, Proteobacteria*, *Verrucomicrobia*, and *Cyanobacteria* [16,17,18]. In particular, the small intestine is predominantly colonized by Gram-positive bacteria belonging to the phylum *Firmicutes*, comprising the classes *Streptococcus*, *Veillonella*, and *Clostridium* [19]. In contrast, the large intestine harbors a much more diverse microbial community, primarily dominated by the phyla *Bacteroidetes*, comprising the class of *Bacteroides*, and *Firmicutes*, which includes the classes *Clostridium*, *Faecalibacterium*, *Lactobacillus*, and *Ruminococcus* [20]. The ratio of *Firmicutes* to *Bacterioidetes* (F/B) is often considered an estimate of gut microbial health. However, the F/B ratio is not the same in all individuals. Dietary habits and other factors (type of delivery, geographic origins, host genetics, age, sex, body mass index (BMI), use of antibiotics or probiotics) shape the composition of gut microbiota, altering this ratio [21]. Moreover, the abundance of *Bacteroidetes*, *Firmicutes*, *Actinobacteria*, and *Proteobacteria* can be influenced by various everyday exposures, including excessive chlorinated water intake, food additives, and environmental contaminants such as mycotoxins, heavy metals, and pesticides [22]. Thus, although the F/B ratio has been historically used as a descriptive marker of gut microbial health, it is increasingly recognized as a nonspecific and context-dependent metric that should be interpreted with caution. Functional profiling and metabolite-based analyses provide more biologically meaningful insights into host–microbiome interactions.

The human body (host) is responsible for the growth and protection of these intestinal microbial communities. This highly complex and symbiotic microbial system, in turn, actively participates in the digestion of macronutrients, synthesizes essential vitamins for metabolic homeostasis, regulates immune system, and forms a barricade against pathogenic bacteria [23,24].

Gut microbiota breaks down otherwise indigestible dietary components. Through the fermentation of complex carbohydrates (fibers and oligosaccharides) and host-derived glycans, microbes produce SCFAs, such as acetate, propionate, and butyrate, which serve as important energy substrates for colonocytes and regulate systemic metabolism [14,25]. SCFA production occurs alongside the conversion of primary bile acids (BAs) into secondary BAs, such as deoxycholic acid and lithocholic acid, through gut microbial action. Primary BAs—cholic acid and chenodeoxycholic acid—are synthetized in the liver from cholesterol [26]. During enterohepatic cycling, primary BAs undergo deconjugation, and some of these (5 to 10%) can then be converted into secondary BAs in the distal intestine through the gut microbial enzymatic activity [26,27]. Once absorbed, secondary BAs modulate lipid signaling pathways and, at least in part, immune functions via interaction with their receptors, including Takeda G protein-coupled BA receptor-1 (TGR5), farnesoid X receptor (FXR), and xenobiotic pregnane X receptor (PXR) [27]. In addition, gut microbes contribute to the biosynthesis of essential vitamins (e.g., vitamin K and B-group vitamins), facilitate the biotransformation of bile acids, and assist in drug and xenobiotic metabolism, thereby influencing absorption and pharmacokinetics [28].

The gut microbiota plays a critical role in the regulation of the host immune system, helping establish immunological tolerance and preventing excessive inflammation. Commensal microbes shape mucosal and systemic immunity by stimulating the production of secretory IgA, promoting T cell differentiation, and modulating inflammatory pathways. For example, *Bacteroides fragilis* produces polysaccharide A, which promotes anti-inflammatory immune signaling via Toll-like receptor 2 (TLR2), thereby enhancing immune tolerance [29]. Gut microbes also offer a barrier effect, inhibiting pathological colonization by occupying ecological niches, competing for nutrients, and producing antimicrobial metabolites [30]. Secondary BAs and bacteriocins generated by the microbiota inhibit the growth of pathogenic species, including *Clostridioides difficile*, thereby protecting the host from infection [30].

Finally, the gut microbiota supports the structural and functional integrity of the intestinal barrier. Microbial metabolites regulate epithelial cell proliferation and mucus secretion, strengthen tight junctions, and protect against barrier disruption. This function is crucial for preventing the translocation of pathogens and endotoxins into systemic circulation, thereby preventing inflammation [31].

### Gut Microbiota in Cardiometabolic Health

Accumulating evidence underscores the crucial role of the gut microbiota in regulating vascular function, BP, lipid and glucose metabolism, and overall cardiometabolic homeostasis through multiple mechanistic pathways. Indeed, gut microbiota has recently been recognized as an important factor in the development and progression of cardiometabolic diseases [32,33]. Numerous studies have reported alterations in both the composition and function of gut bacteria in individuals with cardiometabolic diseases. For instance, both animal and human studies on obesity have revealed gut microbial alterations [34]. Additionally, gut dysbiosis can impair intestinal barrier integrity, disrupt glucose sensitivity and absorption, and ultimately contribute to insulin resistance and T2D [35].

Gut-derived bioactive metabolites pass through the intestinal epithelium, enter the systemic circulation, and subsequently interact with cardiac tissue [36]. Lipopolysaccharides (LPSs) found in the outer membrane of Gram-negative bacteria in the gut can cross the intestinal epithelium and activate immune responses implied in cardiovascular disease progression. Moreover, microbial metabolism generates a variety of bioactive compounds, including SCFAs, trimethylamine-N-oxide (TMAO), branched-chain amino acids, indole and secondary BAs [28,37], which actively interact with cardiovascular functions. Among the metabolites, SCFAs play a fundamental role in maintaining cardiovascular homeostasis and act as potential protective agents against T2D, obesity, and cardiovascular diseases [38,39].

## 3. Short-Chain Fatty Acids: Biology, Metabolism and Functions

### 3.1. Origin and Structure of Short-Chain Fatty Acids

SCFAs are monocarboxylic acids with a hydrocarbon chain of 1 to 6 total carbon atoms, whereas those of 7 to 12 carbon atoms are defined as medium-chain fatty acids (MCFAs), and those with more than 12 carbon atoms are defined as long-chain fatty acids (LCFAs). SCFAs include formate (C1), acetate (C2), propionate (C3), butyrate (C4), and valerate (C5) [40].

SCFAs are produced by colonic anaerobic bacteria through the metabolism of complex resistant carbohydrates like dietary fibers, resistant starch, and some oligosaccharides (e.g., fructo-oligosaccharides, inulin, and polysaccharides from plant cell walls), which escape digestion and absorption in the small intestine and undergo saccharolytic fermentation in the large intestine [41]. It is estimated that the fermentation of approximately 50–60 g of carbohydrates per day leads to the generation of about 500–600 mmol of SCFAs in the gut [42]. Amino acids can also undergo fermentation to yield SCFAs [43]. Collectively, acetate (C2), propionate (C3), and butyrate (C4) account for almost the totality of SCFAs produced by gut bacteria [44]. Overall, in stool and colon, acetate, propionate, and butyrate are found in an approximately molar ratio of 3:1:1 [45], with acetate being the most abundant in the colonic lumen [46]. Concentration of SCFAs in portal blood (375 μmol/L) is nearly five times higher than in peripheral venous blood (79 μmol/L), indicating that the gut serves as the main source of SCFAs [47].

#### 3.1.1. Acetate

Acetate (C2) is generated through two different pathways. Firstly, acetyl-CoA can be generated through the decarboxylation of pyruvate and subsequently hydrolyzed to acetate by the enzyme acetyl-CoA hydrolase [48]. Enteric bacteria, including *Prevotella* spp., *Ruminococcus* spp., *Bifidobacterium* spp., *Bacteroides* spp., *Clostridium* spp., *Streptococcus* spp., *Akkermansia muciniphila*, and *Blautia hydrogenotrophica*, produce acetate though this pathway [49,50]. Secondly, some acetogenic bacteria including *Clostridium* spp. and *Streptococcus* spp. can generate acetate through the reductive methylation of CO_2_ via the Wood–Ljungdahl pathway [48,51]. After production, acetate is quickly absorbed into the bloodstream of the host via diffusion and monocarboxylate transporter activity. Once in circulation, it is taken up by the liver [47], where is serves as a precursor for cholesterol synthesis and lipogenesis [46,52,53].

#### 3.1.2. Propionate

Although propionate-producing bacteria are found across several phyla, only a limited number of genera can synthesize this SCFA [54]. Propionate (C3) can be generated via three main biochemical pathways: the succinate, acrylate, and propanediol pathways [54].

In the succinate pathway, propionate is generated using the primitive electron transfer chain using phosphoenolpyruvate (PEP) [55]. PEP is first carboxylated to oxaloacetate, which is then converted to malate and fumarate. Fumarate serves as an electron acceptor for NADH through the actions of fumarate reductase and NADH dehydrogenase, forming a simple electron-transfer chain. The NADH dehydrogenase pumps protons across the cell membrane, enabling chemiosmotic ATP synthesis. Succinate is produced via fumarate reductase activity. When the partial pressure of carbon dioxide is low, succinate is converted to methylmalonate, which subsequently forms propionate and carbon dioxide [43]. The latter is recycled for PEP carboxylation and continues the cycle. This pathway is used by *Bacterioidetes* and several *Firmicutes* species within the *Negativicutes* class [43].

In the acrylate pathway, lactate is reduced to propionate via lactoyl-CoA dehydratase [48,54]. This route is present in only a few gut bacteria, including *Coprococcus catus* [54], *Clostridium propionicum*, and *Megasphaera elsdenii* [45,54].

Finally, in the propanediol pathway, 1,2-propanediol derived from deoxy sugars such as rhamnose and fucose, is converted sequentially to propionaldehyde and propionyl-CoA, leading to propionate formation [56]. *Roseburia inulinivorans* [57] and *Akkermansia muciniphila* [58], which appears to be one of the major propionate-producing species, utilize this pathway.

Like acetate, propionate is rapidly absorbed into the host bloodstream via passive diffusion and monocarboxylate transporter-mediated uptake. Upon entering systemic circulation, it is primarily taken up by the liver [47], where it serves as a substrate for protein synthesis, lipogenesis, and gluconeogenesis [59].

#### 3.1.3. Butyrate

Butyrate (C4) is mainly produced by members of the *Ruminococcaceae* and *Lachnospiraceae* families [60]. Fermentation of resistant starch plays a major role in generating butyrate in the colon, with *Ruminococcus bromii* recognized as a key contributor, and its absence has been linked to reduced resistant starch fermentation [61].

Butyrate generation begins with the condensation of two acetyl-CoA molecules into acetoacetyl-CoA. Acetoacetyl-CoA is then sequentially reduced to β-hydroxybutyryl-CoA, crotonyl-CoA, and finally butyryl-CoA [62]. From butyryl-CoA, butyrate can be synthesized through two distinct pathways. In *Coprococcus* spp. [63], the enzymes phosphotransbutyrylase and butyrate kinase catalyze the conversion of butyryl-CoA to butyrate [43]. Conversely, in bacteria including *Faecalibacterium prausnitzii*, *Eubacterium rectale*, *Eubacterium hallii*, and *Ruminococcus bromii*, the butyryl-CoA:acetate CoA-transferase pathway converts butyryl-CoA into butyrate and acetyl-CoA by utilizing exogenously derived acetate [64].

Butyrate acts as fuel for colonocytes, being absorbed only in minimal amounts [53]. Consequently, under normal physiological conditions, circulating levels of acetate and propionate are substantially higher than those of butyrate.

### 3.2. Effects of SCFAs on Cardiac Metabolism

The principal fuel for oxidative adenosine triphosphate (ATP) production in cardiomyocytes is provided by LCFAs, which are energy-rich molecules, reaching up to 70% of the carbon entering the tricarboxylic acid cycle [65]. Other sources of ATP for the heart are represented by glucose, ketone bodies, and lactate [66,67]. Cardiomyocytes in cardiometabolic HFpEF exhibit metabolic inflexibility and a preferential oxidation of fatty acids and ketone bodies rather than glucose, typically occurring under conditions of insulin resistance and mitochondrial dysfunction [67,68,69]. However, HFpEF is markedly characterized by a reduction in LCFA oxidation at the same time, which can decline by 30% [70,71]. This impairment is largely attributable to decreased activity of carnitine palmitoyltransferase I (CPT1), the rate-limiting enzyme for LCFA oxidation located on the outer mitochondrial membrane [69,72]. Due to their short aliphatic chain, SCFAs are less efficient than LCFAs as cardiac energy substrates. However, their CPT1-independent transport into mitochondria may offer an advantage when this rate-limiting enzyme is pathologically downregulated [45,73]. Although SCFAs require a carrier—the monocarboxylate transporter (MCT)—to enter the cytoplasm, once inside the cell, they can diffuse freely into mitochondria. Moreover, in hypertrophic failing heart induced in rats by transverse aortic constriction (TAC), SCFAs proved to be more readily oxidized compared to ketones, despite both bypassing reduced CPT1 activity [74]. This casts doubts on the concept of ketone bodies as a unique potential therapeutic “supefuel” for the failing heart [74]. In a porcine model of coronary artery stenosis, the hypoperfused myocardium showed a preference for oxidizing SCFAs rather than LCFAs, likely due to their ability to bypass the CPT1 inhibition induced by moderate tissue hypoxia, confirming the cardiac metabolic potential of SCFAs [73].

In summary, SCFAs may represent an alternative mitochondrial fuel in the failing heart. In HFpEF, impaired LCFA oxidation and reduced CPT1 activity may limit mitochondrial energy production. SCFAs may support myocardial energy homeostasis through MCT-dependent uptake and CPT1-independent mitochondrial entrance. For this reason, SCFAs represent an unexplored carbon source for energy production in failing hearts (Table 1). Notably, most evidence supporting SCFAs as alternative myocardial fuels comes from animal or *ex vivo* models. The translational relevance of these findings in human HFpEF remains to be established.

### 3.3. SCFAs, Lipid Metabolism and Glucose Homeostasis

SCFAs lower plasma lipid levels by inhibiting lipolysis and promoting lipogenesis [75,76,77]. Among the SCFAs, acetate and propionate can suppress endogenous lipolysis, while propionate also modulates extracellular lipolysis by enhancing lipoprotein lipase expression. Both mechanisms contribute to reduced circulating lipid levels and body weight [75]. Moreover, SCFAs stimulate adipocyte differentiation [78,79]. Treatment of preadipocytes with propionate and acetate has been shown to promote adipocyte differentiation through the upregulation of G protein-coupled receptor 43 (GPR43 or free fatty acid receptor-2, FFAR-2) and peroxisome proliferator-activated receptor γ (PPARγ) [78,79]. Additionally, SCFAs have been reported to increase hepatic cholesterol uptake from the bloodstream, thereby lowering plasma cholesterol levels in animal models [80,81]. Moreover, in preclinical models, propionate acts as a potent inhibitor of cholesterol synthesis [82] and helps reduce plasma cholesterol by increasing its hepatic uptake [81].

Multiple studies have indicated that SCFAs may improve glucose homeostasis in vivo by regulating blood glucose levels and promoting glucose uptake through the activation of G protein-coupled receptor 41 (GPR41 or free fatty acid receptor-3, FFAR-3) and GPR43 [83,84,85]. Although the underlying mechanisms remain not fully elucidated, these effects may occur directly via an AMP-activated protein kinase (AMPK)-dependent coregulatory pathway that involves PPARγ-mediated regulation of gluconeogenesis and lipogenesis [86]. Studies on preclinical models demonstrate that butyrate influences glucose metabolism by upregulating AMPK-dependent gene expression [87], while propionate reduces hepatic gluconeogenesis through the same mechanism [88]. In a high-fat diet-induced model of metabolic syndrome, SCFA administration protected against obesity, insulin resistance, and hepatic steatosis, primarily through the inhibition of PPARγ [46]. Additionally, propionate enhances glucose-stimulated insulin secretion through GPR43 activation and increasing β-cell proliferation [83].

Under normal conditions, SCFAs regulate food intake by promoting the secretion of satiety hormones such as peptide YY (PYY) and glucagon-like peptide 1 (GLP-1) through activation of GPR41 and GPR43 [89,90], as well as through the inhibition of histone deacetylases (HDACs) [91]. PYY can facilitate glucose clearance in adipose tissue and muscle, whereas GLP-1 enhances insulin secretion and suppresses glucagon release from the pancreas, thereby contributing to blood glucose regulation [89]. Moreover, it has been demonstrated that acetate can cross the blood–brain barrier and suppress appetite [92].

Although SCFAs exert many beneficial effects, their actions are highly context dependent. For instance, acetate acts as a signaling molecule that improves glucose and lipid metabolism in white adipose tissue (WAT) via GPR43 stimulation [113]. However, it also serves as a direct metabolic substrate for lipogenesis and cholesterol synthesis. Thus, when acetate availability is elevated—such as during excess caloric intake or increased colonic production—it can stimulate lipogenic activity that may exacerbate hepatic steatosis in the context of cardiometabolic diseases. Consequently, beneficial signaling effects may coexist with excessive lipogenesis, underscoring dose–response relationships and trade-offs between metabolic regulation and lipid synthesis that are highly dependent on dietary context and metabolic health.

In summary, SCFAs modulate lipid and glucose homeostasis through the interaction with G protein-coupled receptors (GPR41 and GPR43), and AMPK- and PPARγ-related pathways. The ability to control lipid turnover and glucose utilization, along with control of appetite, underscores their potential as metabolic mediators in modulating cardiometabolic homeostasis (Table 1). However, these effects are highly context dependent, underscoring that SCFAs should not be viewed as uniformly beneficial, but rather as metabolic modulators whose beneficial effects depend on doses, timing, and underlying cardiometabolic context. Further research is therefore required to clarify context-specific effects, dose–response relationships, and potential metabolic trade-offs associated with SCFA exposure.

### 3.4. Effects of SCFAs on Myocardial Contractility, Blood Pressure, and Heart Rate

SCFAs have also been demonstrated to exert non-metabolic functions, acting as signaling molecules, and regulating myocardial contractility, BP, and activity of the autonomic nervous system.

Butyrate has been reported to improve myocardial stunning by enhancing contractility in the post-ischemic heart [93]. Additionally, evidence from human studies highlights a strong association between increased SCFA levels and lower BP [96,97]. Gut-derived SCFAs interact with GPR41 to regulate BP [53]. Indeed, knock-out GPR41 mice were demonstrated to have hypertension of vascular origin [98]. GPR41 receptors are located on smooth muscle and endothelial cells of low-resistance blood vessels, and their stimulation promotes vasodilation and BP reduction [98,99,100,101]. SCFAs also bind olfactory receptor-78 (Olfr78 in mice or OR51E2 in humans), which might play an opposing role in the regulation of BP, balancing the effects of GPR41 binding [100].

SCFAs have been demonstrated to exert antihypertensive effects in multiple experimental animal models. Consistently, in preclinical models of angiotensin II-induced hypertension, administration of acetate, propionate, or butyrate significantly reduced BP, cardiac hypertrophy, and fibrosis [94]. In these models, the antihypertensive effect of SCFAs was linked to GPR-dependent reductions in peripheral resistance and decreased renal sodium/potassium excretion ratio [94]. Additional experimental animal studies have confirmed the BP-lowering properties of SCFAs [114,115]. Moreover, in mice models of isoproterenol (ISO)-induced cardiac hypertrophy, acetate improved overall cardiac functions and decreased myocardial fibrotic alterations [95].

SCFAs are also implied in the regulation of autonomic nervous system activity [95]. In the gut, butyrate-mediated stimulation of GPR41 and GPR43 enhances vagal tone, resulting in reduced heart rate and hypotension [102].

In summary, SCFAs exert multifaceted cardiovascular effects by modulating myocardial contractility, BP, and autonomic nervous system activity through direct GPR-mediated signaling (Table 1). These findings underscore their potential role in the management of patients with HFpEF.

### 3.5. Effects of SCFAs on Inflammation and Immunomodulation

Strong evidence supports a critical role of SCFAs in modulating inflammation and immunity. SCFAs interact with hydroxycarboxylic acid receptor 2 (or GPR109A) [94,116], highly expressed on neutrophils, monocytes, and macrophages [117,118]. The activation of GPR109A receptor by butyrate triggers an anti-inflammatory cascade via inhibition of the NF-κB pathway [103]. Moreover, the binding of SCFAs with GPR43 and GPR109A receptors modulates the NLR family pyrin domain-containing 3 (NLRP3) inflammasome exerting anti-inflammatory effects [104]. NLRP3 inflammasome mediates the inflammatory response in HF and cardiometabolic HFpEF [105,106]. SCFAs have also been shown to inhibit the differentiation of pro-inflammatory Th1 and Th17 cells while promoting both the differentiation and suppressive function of anti-inflammatory Treg cells [107,108]. The anti-inflammatory action of SCFAs has also been demonstrated in the myocardium of rat models of angiotensin II-induced hypertension, where oral butyrate suppressed cyclooxygenase-2 and prostaglandin E2 expression, leading to reduced atrial natriuretic peptide production [109].

In conclusion, by modulating pro-inflammatory pathways and NLRP3 inflammasome and promoting regulatory immune responses, SCFAs may exert potent immunomodulatory effects that directly counteract the systemic inflammation central to the pathophysiology of cardiometabolic HFpEF (Table 1).

### 3.6. SCFAs and Epigenetic Modulation

SCFAs exert other effects through epigenetic mechanisms, particularly by regulating histone post-translational modifications (PTMs). Histones, around which DNA is wound to form compact chromatin, undergo various PTMs such as acetylation, methylation, and ubiquitination [119]. These modifications influence chromatin structure, DNA accessibility, and resulting transcription [119]. Numerous enzymes finely regulate histone PTMs, including histone deacetylases (HDACs), which catalyze the removal of acetyl groups [120]. To date, in mammals, 18 HDACs have been identified and categorized in four classes. Class I HDACs, including HDAC1, 2, 3, are inhibited by acetate [121], propionate, and particularly butyrate [122], preventing histone deacetylation [123]. Whether this inhibition occurs directly or via GPR interaction remains unclear [124,125]. Functionally, inhibition of HDAC-mediated deacetylation reduces monocyte production of TNFα and NF-κB, thereby exerting anti-inflammatory effects [110]. Moreover, evidence from preclinical models shows that a high-fat diet markedly increases intestinal HDAC3 expression, which, in turn, enhances the expression of fatty acid transporters such as CD36, and suppresses enzymes that are critical for fatty acid oxidation, such as CPT1 [111]. Inhibition of intestinal HDAC3 in mouse models prevents lipid absorption and lowers the risk of weight gain, dyslipidemia, and diabetes [111,112]. Butyrate strongly inhibits HDAC3 [112], thereby holding therapeutic promise for metabolic syndrome and its complications, including HFpEF. Similarly, butyrate inhibits class II HDACs, including HDAC5 and HDAC6, downregulating pro-inflammatory genes such as IL-1β, NLRP3, and MCP-1, contributing to anti-inflammatory and BP-lowering effects [109]. SCFA-induced epigenetic modulation may also influence DNA methylation. SCFA-driven effects on DNA methylation promote regulatory T-cell activation, reinforcing their immunoregulatory role [69].

In summary, the inhibition of HDAC-mediated deacetylation and regulation of DNA methylation by SCFAs may contribute to improved metabolic homeostasis and reduced inflammation, highlighting their potential for cardiometabolic HFpEF (Table 1).

## 4. Gut Microbiota and SCFAs in HFpEF

Emerging research highlights the pivotal influence of the gut microbiota composition in cardiovascular homeostasis, acting through multiple mechanistic pathways. Dysbiosis impairs gut barrier function, BP regulation, lipid and glucose metabolism, and overall cardiometabolic health [126,127].

Although increasing evidence supports a pivotal role of gut microbiota alterations in cardiometabolic diseases, data specifically addressing cardiometabolic HFpEF remain limited. Conversely, substantial experimental evidence underlines the possible role of gut microbial dysbiosis in cardiometabolic conditions that are critical for HFpEF, such as hypertension, obesity, and T2D, all conditions that are known to promote systemic inflammation, endothelial dysfunction, and metabolic derangements. Recent advances in genome sequencing technologies and bioinformatics have made it possible to identify gut microbial composition and functions, as well as specific microbial signatures and functional changes, leading to an intensive investigation of potential roles of gut microbes in the pathogenesis of cardiometabolic disorders [34,128,129,130].

### 4.1. Gut Microbiota Composition in Cardiometabolic Comorbidities of HFpEF

Across cohorts of hypertensive patients, gut microbiota composition is characterized by a decrease in α-diversity [131], which is a quantitative measure of community diversity [132] and alterations in **β**-diversity [133], a measure of similarity between stool samples of different patients [132]. Gut dysbiosis in hypertension is primarily marked by a depletion of SCFA-producing bacteria such as *Faecalibacterium*, *Roseburia*, *Ruminococcus*, *Bifidobacterium*, *Akkermansia*, and *Bacteroides*, accompanied by an overgrowth of potentially pro-inflammatory taxa including *Klebsiella*, *Prevotella*, *Enterobacter*, *Megasphaera*, and *Alistipes* [131,134,135]. These compositional changes lead to diminished SCFA production, weakening vasodilatory and immunoregulatory signals. A systematic review of 17 studies on hypertensive patients [131] showed increased fecal but decreased plasma SCFA levels, indicating impaired intestinal absorption and excessive excretion, which may contribute to BP dysregulation through reduced SCFA-mediated signaling via GPRs. Additionally, functional analyses revealed upregulation of pathways involved in LPS biosynthesis, suggesting a shift toward pro-inflammatory and metabolically imbalanced gut microbial activity [131]. Evidence in experimental models of hypertension revealed a decrease in the expression of tight junction proteins, which suggests a breakdown of the gut epithelial barrier [136]. Therefore, epithelial cells are exposed to the gut microbiota and their metabolites, which exacerbates immune activation and the secretion of pro-inflammatory factors [136].

Evidence indicates that gut microbial composition also plays a role in obesity and T2D. Human studies revealed compositional differences between obese and lean individuals. Obesity is characterized by elevated fecal SCFA concentrations [137,138], despite reduced circulating SCFAs [139]. Specifically, obese individuals tend to have decreased levels of butyrate-producer bacteria [140]. Similarly, growing evidence links gut microbiota dysbiosis with glucose metabolism abnormalities in prediabetes and T2D. Mechanistic studies suggest that hyperglycemia itself can increase intestinal permeability via GLUT2-mediated transcriptional changes and disruption of tight junctions, creating a “leaky gut” that may promote systemic inflammation and insulin resistance [141]. In drug-naive individuals with prediabetes, the gut microbiota typically exhibits a loss of butyrate-producing taxa, reduced *Akkermansia muciniphila* and increased abundance of bacteria with pro-inflammatory properties [142,143]. T2D is associated with a decline in butyrate-producing bacteria [130]. Overall, studies in obesity and T2D consistently indicate their strong association with gut microbial dysbiosis characterized by a loss of beneficial butyrate producers, decreased microbial diversity, and enrichment of pro-inflammatory and metabolically detrimental taxa [28].

**Figure 1:**
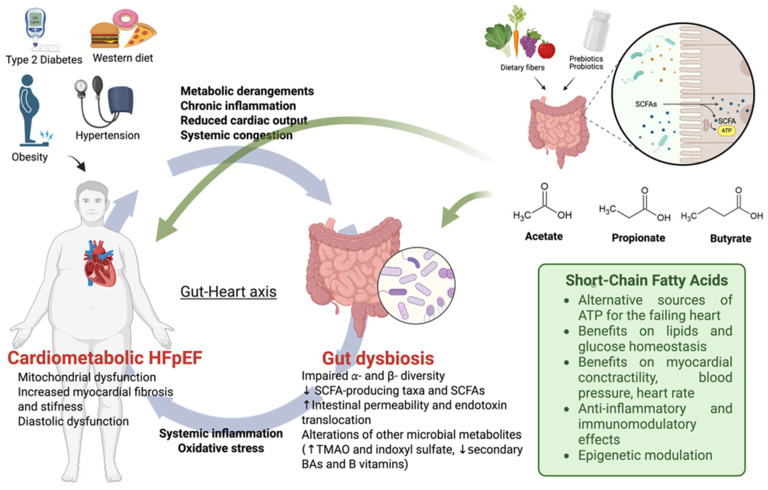
Hypothesis-generating conceptual framework of the gut–heart axis and SCFAs in cardiometabolic HFpEF. Cardiometabolic HFpEF may promote gut dysbiosis through metabolic derangements, chronic inflammation, reduced cardiac output, and systemic congestion. Gut dysbiosis is characterized by reduced SCFA-producing taxa in gut microbiota composition and decreased SCFA production, increased permeability, endotoxin translocation, and alteration of other microbial metabolites. These alterations are associated with systemic inflammation and oxidative stress, which may contribute to mitochondrial dysfunction of cardiomyocytes, increased myocardial fibrosis, stiffness, and diastolic dysfunction. SCFAs and restoration of their homeostasis through nutritional interventions may represent a novel therapeutic avenue in cardiometabolic HFpEF, potentially exerting favorable effects on cardiac energy metabolism and function, lipid and glucose homeostasis, inflammatory, immune, and epigenetic modulation. Created in BioRender. Sechi, L. (2026) https://BioRender.com/9kkqybo.

Collectively, these findings underscore the central role of gut microbial dysbiosis as a shared pathological feature across common comorbidities of cardiometabolic HFpEF. The consistent loss of beneficial SCFA-producing taxa increases intestinal permeability with an enrichment of pro-inflammatory microbial pathways (Figure 1). This suggests that impaired gut barrier integrity and altered metabolite signaling may form a common mechanistic axis, potentially linking gut microbiota to cardiometabolic dysfunction.

### 4.2. Gut Dysbiosis in HFpEF

While the role of the gut microbiota in cardiometabolic disorders is well-investigated, studies focusing on changes in gut microbiota composition in HFpEF is limited. To date, few studies have focused on this topic. Moreover, available studies are limited by small sample sizes, heterogeneous phenotyping, older cohorts, and residual confounding by diet, comorbidities, and medications.

In a study involving 26 patients with HFpEF and 67 control participants from two independent cohorts [144], the gut microbiota was found to be profoundly altered in HFpEF compared with both metropolitan and regional healthy controls. Patients with HFpEF demonstrated significant differences in β-diversity and α-diversity, indicating decreased microbial richness. These alterations were independent of confounding factors, including age, BMI, hypertension, sex, and dietary fiber intake. Taxonomic analysis revealed a selective depletion of SCFA-producing taxa, particularly *Ruminococcus*, which is involved in the generation of butyrate and other SCFAs through the fermentation of dietary fiber. Moreover, patients with HFpEF exhibited lower dietary fiber intake and reduced overall diet quality, which may contribute to impaired SCFA production and consequent systemic inflammation, endothelial dysfunction, and myocardial fibrosis. These findings suggest that gut microbial dysbiosis, characterized by loss of SCFA-producing bacteria and reduced diversity, is associated with HFpEF and may contribute to its development and progression. Another team analyzed gut microbiota composition in 30 patients with HFpEF compared to 30 healthy controls [145]. In this study, high-throughput DNA sequencing analyses revealed reduced gut microbial richness (lower α-diversity) and compositional shifts (altered β-diversity) in patients with HFpEF, including increased abundance of pro-inflammatory taxa and depletion of anti-inflammatory genera such as *Butyricicoccus*, *Lachnospira*, and *Ruminiclostridium*. These beneficial taxa are typically involved in SCFA production, intestinal barrier maintenance, and immune modulation. Overall, HFpEF-associated dysbiosis may sustain low-grade systemic inflammation, contributing to disease severity and poor prognosis (Figure 1).

Importantly, the interpretation of HFpEF-specific microbiome studies warrants caution. Available studies are limited by small sample sizes, heterogeneous phenotyping, residual confounding by diet, comorbidities, and medications. Moreover, it is important to consider potential selection and collider bias in HFpEF cohorts, which often include older, multimorbid cohorts. In addition, microbiome studies face several methodological challenges that limit reproducibility, including batch effects, variability in DNA extraction protocols, differences in sequencing depth and normalization strategies, and the compositional nature of microbiome data. The lack of standardized analytical pipelines further complicates reproducibility and causal inference.

#### 4.2.1. Disruption of Intestinal Barrier and Microbial Translocation

Evidence consistently reports disruption of intestinal barrier always observed in HF progression [146,147]. The “gut hypothesis of heart failure” suggests that reduced cardiac output and increased systemic congestion causes intestinal mucosal ischemia and/or edema, promoting the disruption of the epithelial barrier with increased intestinal permeability [148]. Gut microbiota participates in regulating the homeostasis of the intestinal barrier, which comprises mechanical, chemical, immunological, and biological components that collectively maintain gut integrity [149]. In HFpEF, the stability of the gut microbiota is disrupted and impairs intestinal colonization resistance, facilitating the invasion and proliferation of potentially pathogenic organisms, including opportunistic bacteria. Therefore, bacterial translocation leads to the passage of intestinal microbiota and their components, such as endotoxins, peptidoglycans, or metabolites, across the intestinal mucosa into extraintestinal tissues and organs [150]. Higher levels of pathogenic microbes such as *Candida*, *Campylobacter*, *Salmonella*, *Shigella*, and *Yersinia enterocolitica* are reported in patients with chronic HF compared with healthy individuals [151]. Plasma endotoxin and cytokine concentrations also appear elevated in these patients, confirming bacterial translocation [152,153]. Even in HFpEF, the disruption of tight junctions between intestinal epithelial cells increases intestinal permeability, potentially facilitating metainflammation [13] (Figure 1).

#### 4.2.2. Gut Dysbiosis and Systemic Inflammation

A growing body of experimental and clinical evidence highlights the pivotal role of inflammation in the onset and progression of cardiometabolic HFpEF, which arises from systemic perturbations [7,154]. Irrespective of the underlying etiology, HF is closely associated with both local and systemic inflammatory responses [155]. However, patients with HFpEF exhibit higher systemic inflammatory burden compared with patients with heart failure with reduced ejection fraction (HFrEF). Indeed, circulating concentrations of inflammatory biomarkers, such as tumor necrosis factor (TNF)-α, interleukin (IL)-6 and IL-8, high-sensitivity C-reactive protein (hsCRP), pentraxin-3, and chemokine (C–C motif) ligand 2 (CCL2) are significantly elevated in HFpEF compared with HFrEF or other cardiac disorders [6,156]. Elevated levels of these inflammatory mediators contribute to endothelial dysfunction, myocardial fibrosis, and other pathological remodeling processes that drive HFpEF progression [157,158,159]. Moreover, endomyocardial biopsies from HFpEF patients reveal an increased presence of inflammatory cells expressing the pro-fibrotic cytokine transforming growth factor-β (TGF-β) [160]. NLRP3 inflammasome, which drives the release of IL-1β and IL-18 [105,106], also mediates systemic inflammation and cardiac remodeling in HFpEF [13]. Indeed, its pharmacological inhibition attenuates maladaptive cardiac changes [161]. Activation of the NLRP3 inflammasome has been observed in mouse models of HFpEF [162,163]. Moreover, NLRP3 activation within endothelial cells disrupts intercellular junctions, causing endothelial dysfunction [164]. Gut microbiota-derived metabolites modulate NLRP3 inflammasome activity, thereby amplifying inflammation and myocardial fibrosis [165,166].

Patients with HFpEF exhibit an increased abundance of pro-inflammatory bacteria and a reduction in anti-inflammatory species [145]. Translocation of microbial products and elevation of endotoxin levels into the circulation due to increased intestinal permeability contributes to systemic inflammation [167,168]. A recent study using an animal model of HFpEF (DOCA [deoxycorticosterone acetate] combined with a high-fat diet) reported a reduction in the thickness of the colonic mucus layer, which is increasingly recognized as a key factor for the promotion of inflammation [169]. Indeed, microbial bioactive compounds may cross the intestinal epithelium due to increased gut permeability, thereby reaching the systemic circulation, causing inflammation and affecting cardiac structure and function [126].

Bacterial structural components and metabolites can translocate into the circulation, activating immune responses and triggering inflammatory cascades [170]. LPS, a component of Gram-negative bacteria, can enter the bloodstream and trigger systemic inflammation [171]. LPS binds to lipopolysaccharide binding protein (LBP), which subsequently interacts with soluble CD14 (sCD14) [172,173]. LPS and sCD14 levels may be detected in blood and represent indirect measures of gut permeability [174]. The increase in these biomarkers is associated with worsening of HF [175]. Additionally, increased sCD14 levels are associated with higher mortality in patients with HF [176] and incidence of HFpEF, but not HFrEF [177]. Moreover, LPS activates the host Toll-like receptor 4 (TLR4), contributing to inflammation and cardiac injury [178]. TLR4 enhances NF-κB translocation, leading to transcription of inflammatory markers like IL-6 [179]. In animal models, pharmacological inhibition of TLR4 has been shown to normalize BP and prevent cardiac remodeling [180]. Additionally, LPS can directly impair the paracellular pathway, further increasing intestinal permeability [181]. Pathways related to LPS response are upregulated in HFpEF compared to HFrEF and show an association with increased myocardial fibrosis [182].

In summary, gut microbiota-derived endotoxins and metabolites may contribute to systemic and cardiac inflammation through NLRP3 and TLR4 signaling, supporting the gut–heart axis as a potential driver of metainflammation in HFpEF (Figure 1).

### 4.3. Gut Microbial Metabolites and SCFAs

Metabolites and signaling molecules of gut microbiota, including trimethylamine-N-oxide (TMAO), aromatic amino acid (AAA) derivatives, B vitamins, and BAs, can collectively modulate cardiac function and disease progression (Figure 1). Metabolic connections between these metabolites and SCFAs are essential to understand the intricate contribution of gut microbiota dysregulation in the pathogenesis of HFpEF.

#### 4.3.1. Trimethylamine-N-Oxide

TMAO and SCFAs exhibit opposing effects in HF. Therefore, elevated TMAO levels in patients with HF correlate with decreased SCFA-producing bacteria [183]. Gut microbiota metabolism of choline, phosphatidylcholine and L-carnitine produces trimethylamine (TMA) as a waste product whose production is widespread in *Firmicutes*, *Proteobacteria*, and *Actinobacteria* [184]. This organic compound enters the portal circulation, where it is rapidly oxidized into TMAO by flavin monooxygenase enzymes in the liver and then released into the circulation. TMAO has been shown to act as a pathogenetic modulator of cardiometabolic diseases [185]. Indeed, elevated TMAO levels have been linked to increased risk of atherosclerosis, as well as HF development and poor HF prognosis [126,186,187,188]. Mouse models fed high-choline diets exhibited increased cardiovascular disease (CVD) burden, while interventions targeting the TMAO pathway—from antibiotic suppression to the precursor flavin monooxygenases inhibition—ameliorated these effects [127,186]. Patients with HFpEF show higher plasmatic levels of TMAO than healthy individuals [189]. Moreover, TMAO could be used as a marker for risk stratification in HFpEF [190]. Elevated TMAO levels in CD1 mice, fed with a Western diet—rich in sugar and saturated fat—promoted cardiac inflammation and fibrosis, ultimately impairing diastolic function [191]. A prospective cohort study involving 112 participants with chronic systolic HF reported a positive correlation between TMAO levels and indices of diastolic dysfunction, including mitral E/septal Ea and left atrial (LA) volume index [192]. Mechanistically, TMAO enhances the expression of TGF-β/SMAD3 [193] and TGF-β activates fibroblasts and stimulates type I collagen secretion, promoting myocardial fibrosis [194]. TMAO also upregulates pro-inflammatory gene expression through the NF-κB pathway and activates the NLRP3 inflammasome [195].

In summary, elevated TMAO levels are associated with HFpEF in human studies, while experimental models support a causal role in promoting myocardial fibrosis and inflammation. This suggests that targeting its molecular pathways could represent a potential therapeutic approach.

#### 4.3.2. Aromatic Amino Acids

Aromatic amino acids (AAAs) and SCFA metabolism form a key regulatory network, wherein specific bacterial taxa contribute to both processes. Clinical evidence indicates that disturbances in both SCFA and AAA metabolism adversely influence outcomes in patients with HF [196]. Members of *Actinobacteria*, *Firmicutes*, *Bacteroidetes*, and *Proteobacteria* coordinate SCFA production alongside the metabolism of tryptophan and other AAAs [197,198,199,200]. SCFAs and tryptophan-derived metabolites engage in tightly linked metabolic pathways, jointly modulating host inflammatory, immune, and metabolic responses through multiple mechanisms [201,202]. Indole-3-propionic acid (IPA), a gut microbiota-derived metabolite of tryptophan, exerts cardioprotective effects via AhR-mediated regulation of pathways that modulate energy metabolism, reduce oxidative stress, attenuate fibrosis, and improve diastolic function [185,203]. In a mouse model of HFpEF, IPA supplementation reduced myocardial inflammation and improved diastolic function [204]. Conversely, disruption of these pathways elevates indole, which may be absorbed itself and sulfated in the liver to produce harmful metabolites such as indoxyl sulfate (IS). Increased plasma levels of IS are associated with the progression of HF [205]. IS promotes fibrosis, hypertrophy, and inflammation through MAPK and NF-κB activation [206], although to date, there is no evidence in HFpEF.

#### 4.3.3. B Vitamins

SCFA-producing bacteria within the gut microbiome frequently synthesize B vitamins through interconnected metabolic pathways. This coupling between SCFAs and B-vitamin production constitutes a crucial mechanism by which gut microbes support both microbial community balance and host physiology [207]. *Firmicutes* and *Bacteroidetes* exhibit dual metabolic capacities, coordinating the biosynthesis of SCFAs and B vitamins via shared biochemical routes. For instance, within *Firmicutes*, key SCFA-producers such as *Lachnospiraceae* and *Ruminococcaceae* synthesize complex vitamins including folate (B9) and cobalamin (B12) while maintaining robust SCFA output [207,208,209,210]. This metabolic cooperation is particularly significant in the context of HF, where patients commonly exhibit deficiencies in both SCFAs and B vitamins [211,212,213,214]. The functional synergy between these metabolites extends beyond basic nutritional support. B-group vitamins (B1, B2, B6, and B12), in concert with SCFAs, enhance energy metabolism, mitigate oxidative stress, reduce cardiomyocyte apoptosis, and limit myocardial fibrosis. Collectively, these effects improve cardiac efficiency by promoting vascular function, improving contractility, and supporting overall cardiovascular health [215,216]. In preclinical models of HFpEF induced by a high-fat diet and L-NAME administration, supplementation of vitamin B6 prevents the development of HFpEF acting through the downstream of kinase 3 (DOK3) pathway [217].

#### 4.3.4. Bile Acids

The gut microbiome plays a central role in modulating both SCFA and BA production, forming a tightly interconnected metabolic network that profoundly influences cardiovascular health [218,219,220]. SCFA-producing bacteria within the *Firmicutes* and *Bacteroidetes* phyla also participate in BA metabolism, highlighting their shared metabolic interdependence [221]. SCFAs promote the conversion of cholesterol into BAs by upregulating cholesterol 7α-hydroxylase (CYP7A1) expression [222]. Thus, an impairment in SCFA metabolism also affects BA production. The interaction between SCFAs and BAs is specifically relevant in HF, where these metabolites exert regulatory effects. In a study of 163 participants with metabolic dysfunction-associated fatty liver disease (MAFLD), patients that met the HFpEF criteria displayed significantly lower levels of two secondary BAs, ursodeoxycholic acid and hyocholic acid [223]. Moreover, supplementation of lithocholic acid ameliorated hyperglycemia-induced cardiac hypertrophy in H9c2 cells [224].

## 5. Nutritional Strategies to Modulate SCFAs in Cardiometabolic HFpEF

### 5.1. Therapeutic Rationale of SCFAs Modulation in Cardiometabolic HFpEF

Collectively, gut microbiota dysbiosis, reduction in SCFA-producing taxa, and increase in pro-inflammatory taxa are key contributors to the metainflammation of HFpEF. Moreover, the Western diet—associated with HFpEF and composed of high saturated fat and sugar and low dietary fibers—is linked to decreased SCFA concentrations, gut dysbiosis and increased inflammation [225,226,227]. These alterations actively contribute to disease progression, characterized by reduced concentrations of acetate, propionate, and butyrate and increased pro-inflammatory compounds like TMAO. This imbalance systemically amplifies inflammation and oxidative stress and, at the cardiac level, myocardial remodeling and fibrosis. Therefore, these findings provide a strong translational rationale for targeting the gut–heart axis through dietary interventions in cardiometabolic HFpEF. Modulation and enhancement of SCFA production via tailored nutritional strategies represents a physiologically grounded and low-risk approach to attenuate inflammation and improve cardiac functions and metabolism (Table 2). However, it must be considered that the diversity and intrinsic complexity of the gut microbiota across individuals limits the reproducibility of its modulation, thereby positioning diets, probiotics, and prebiotics as adjunctive rather than primary therapeutic options. Furthermore, most interventional evidence to date relies on surrogate endpoints (e.g., microbiota composition, SCFA levels, metabolic biomarkers) rather than specific clinical outcomes like diastolic function, exercise capacity, or symptom burden. Moreover, it is important to emphasize that most mechanistic insights linking SCFAs to myocardial metabolism, inflammation, and fibrosis are derived from experimental and preclinical evidence, while human interventional data in cardiometabolic HFpEF remain scarce and largely indirect. Therefore, current evidence supports SCFA pathways primarily as biologically plausible modulators rather than established therapeutic targets. Thus, nutritional and microbiota-directed strategies should be regarded as adjunctive approaches that may complement, but not substitute, established pharmacological therapies.

Importantly, HFpEF encompasses multiple phenotypes. Therefore, the relevance of gut microbiota-derived SCFAs is likely greatest within the cardiometabolic HFpEF phenotype, where metabolic dysfunction and systemic inflammation represent dominant drivers of disease pathophysiology.

The following sections discuss current nutritional and microbiota-directed interventions aimed at restoring SCFA homeostasis and potentially mitigating cardiometabolic dysfunction in HFpEF.

### 5.2. High-Fiber Diets

Although studies on this topic show some heterogeneity, there is broad consensus that fiber-rich diets enhance the production of SCFAs [228]. Dietary intake of fibers and fiber-rich diets has been reported to significantly increase several SCFA-producer spp., including *Lachnospira*, *Akkermannsia*, *Bifidobacterium*, *Lactobacillus*, *Ruminococcus*, *Roseburia*, *Clostridium*, *Faecalibacterium*, and *Dorea* [229,230,231,232]. Diets high in fibers, such as the Mediterranean, vegetarian, and vegan diets, have been associated with favorable changes in the gut microbiome such as higher α-diversity and specific microbial populations, reductions in inflammatory markers such as c-reactive protein (CRP) and elevated SCFA levels [233,234]. Indeed, a cross-sectional study involving 31 healthy participants found that individuals who adhered to a Mediterranean diet for six months exhibited higher fecal concentrations of propionate and butyrate, as well as increased levels of *Bifidobacterium* and *Faecalibacterium*, compared with those consuming a diet lower in fiber [235]. In individuals with T2D, a high-fiber diet has been shown to reduce glycated hemoglobin (HbA1c) levels and increase glucagon-like peptide-1 (GLP-1) secretion, *Bifidobacteria* counts, and total SCFA production [236]. In a randomized trial including 81 patients with T2D, a fiber-rich diet for three months led to an increase in the SCFA-producing bacteria *F. prausnitzii* and *A. muciniphila*, along with reductions in glucose, total and LDL cholesterol, free fatty acids, and HbA1c levels [237]. These findings suggest that long-term adherence to a high-fiber diet may promote the growth of SCFA-producing bacteria, thereby improving dyslipidemia, glycemic control, and inflammation. In another study on patients with ischemic heart disease, a vegetarian diet for four weeks increased proliferation of the bacterial families *Ruminococcaceae*, *Lachnospiraceae*, and *Akkermansiaceae*, along with reductions in LDL cholesterol, total cholesterol, and body weight compared with those adhering to a meat-based diet [238].

To date, interventional studies specifically targeting dietary modification in HFpEF are lacking. Available data focus primarily on surrogate endpoints such as SCFA levels, inflammatory biomarkers, and metabolic endpoints while evidence for direct improvements in HFpEF clinical endpoints remains limited. Preclinical data on a murine model of HFpEF demonstrated that supplementation with both fiber and acetate resulted in reduced BP, left ventricular hypertrophy, and fibrosis compared with control mice [115]. Recent data from an observational analysis indicated that a higher consumption of fiber-rich foods was associated with more favorable left ventricular structure and function [239]. An ongoing clinical trial (NCT05236413) is investigating the impact of a high-fiber diet combined with high-intensity interval training on clinical and functional outcomes in patients with HFpEF, as well as their grade of gut mucosal health and inflammation.

### 5.3. Omega-3-Rich Diets

Diets rich in omega-3 polyunsaturated fatty acids (PUFAs), particularly eicosapentaenoic acid (EPA) and docosahexaenoic acid (DHA), have been linked to an increased abundance of SCFA-producing bacteria such as *Bifidobacterium* and *Lactobacillus* [240].

Supplementation with omega-3 PUFAs in *Salmonella*-infected mice markedly elevated the SCFA levels, leading to gut microbiota modulation and enhanced host resistance to pathogenic infection [241]. In another experiment on mice, an omega-3 deficient diet led to reduced fecal acetate and butyrate levels [242].

One case report highlighted a significant rise in butyrate-producing bacteria—including *Eubacterium*, *Roseburia*, *Anaerostipes*, and *Coprococcus*—after two weeks of a daily consumption of an omega-3-enriched diet (containing 600 mg of omega-3 [243]). In a randomized trial, 22 healthy middle-aged participants received omega-3 PUFAs capsules or drinks for two months to assess the impact on gut microbiota composition [244]. Results showed higher levels of SCFA-producing genera such as *Lachnospira*, *Roseburia*, *Lactobacillus*, and *Bifidobacterium* in subjects consuming either or both formulations [244]. Other evidence suggests that a daily intake of 4 g of mixed omega-3 PUFAs (DHA and EPA) significantly increases the density of butyrate-producing genera, including *Blautia*, *Bacterioides*, *Roseburia*, and *Coprococcus* [245]. However, these studies largely report changes in microbiota composition without demonstrating improvements in specific clinical outcomes. Moreover, although omega-3 PUFAs promote the growth of SCFA-producing bacterial taxa, the presence of fermentable fibers is essential for SCFA synthesis. Therefore, in HFpEF, the combination of omega-3 PUFAs with dietary fibers may synergistically increase the SCFA levels, improving cardiometabolic health.

### 5.4. Prebiotics

Prebiotics are non-digestible biological compounds that can be selectively utilized by host microorganisms as substrates to foster their growth, conferring health benefits [246,247]. All prebiotics are types of dietary fiber, although not every fiber functions as a prebiotic. To classify a food ingredient as a prebiotic, it must resist digestion, including gastric acidity, enzymatic hydrolysis, and absorption in the upper gastrointestinal tract, then it is fermented by the intestinal microbiota and selectively promotes the growth and/or activity of beneficial intestinal bacteria [248]. Dietary fibers are polysaccharides containing at least 10 monomeric units (MUs) or oligosaccharides comprising 3–9 MUs. Among the polysaccharides, the main subgroups are non-starch polysaccharides (NSPs) and resistant starch (RS), whereas oligosaccharides include resistant oligosaccharides (ROs) [229].

Sources of RS are cereals, potatoes, beans, legumes [249], and RS has been associated with an increase in SCFA production in in vivo and in vitro studies [250,251]. Moreover, the fermentation of food increases the RS content [252]. A systematic review of 39 trials, including patients with healthy conditions and metabolic disorders, evidenced increased SCFA production following RS supplementation [253]. In mouse models of diabetic cardiomyopathy (DCM), RS supplementation improved myocardial fibrosis and hypertrophy, and these findings were consistent with increased SCFA content [254]. An ongoing pilot study (NCT06337812) is currently evaluating the effects of administering 20 g of potato starch, containing 65% RS characterized by high fermentability, twice daily in patients with HFpEF. The primary endpoint focuses on assessing changes in stool and serum SCFA levels.

Among the RO, arabinoxylan oligosaccharides (AXOSs) produced from cereals have shown promise in obesity-induced mice with related metabolic disorders [255]. An intervention trial investigated the effects of daily supplementation of 3 and 10 g/day of wheat bran extract (WBE) containing AXOSs (2.4 and 8 g/day, respectively) for three weeks to 57 healthy subjects [256]. Gas chromatography analyses revealed significant increases in total SCFAs, including acetate, propionate, and butyrate at the higher dose compared to the placebo. Additionally, propionate levels increased even at the 3 g/day dose. Fluorescence in situ hybridization (FISH) analysis indicated a higher abundance of *Bifidobacteria* at the 10 g/day dose [256]. Comparable outcomes have been reported when AXOSs are delivered via fiber-enriched foods [257,258].

Inulin-type fructans (ITFs), including two ROs (short-chain fructooligosaccharides [scFOS] and oligofructose) and the NSP inulin, are fibers with prebiotic potential due to their ability to be selectively utilized by the gut microbiota increasing SCFA levels [259]. These compounds are found in artichokes, asparagus, bananas, chicory root, garlic, onions, leeks, and wheat [260]. ITF consumption is associated with an increase in *Faecalibacterium prausnitzii* and *Ruminococcus*, both of which are butyrate-producing taxa [259]. In a randomized trial of 61 healthy adults, supplementation of inulin for one week increased the plasma levels of SCFAs [261]. An interventional trial on healthy subjects, supplemented with 12 g of inulin daily for four weeks, identified specific inulin-induced changes in fecal microbiota composition with relative abundances of *Anaerostipes* and *Bifidobacterium* [262], which are associated with increased SCFA production [263,264].

In conclusion, prebiotics such as RS, AXOSs, and ITFs show promise in modulating the gut microbiota and enhancing SCFA production. However, interventions to date have shown promising effects on microbiota composition and SCFA levels but have not yet been linked to clinical improvements. The therapeutic potential of prebiotics in cardiometabolic conditions, including HFpEF, remains to be tested in future clinical investigations.

### 5.5. Probiotics

Probiotics are non-pathogenic live microbes that provide health benefits to the host when administered in adequate quantity. Certain probiotics may directly modulate disrupted processes in HF; however, evidence supporting the specific benefits of probiotics in HFpEF is currently lacking.

Probiotics comprise *Saccharomyces boulardii* yeast and lactic acid bacteria, such as *Lactobacillus* and *Bifidobacterium* spp. [265,266,267]. *Saccharomyces boulardii* administration is associated with an increase in fecal SCFA concentrations, although it is not directly involved in their production [268]. In a randomized trial on 20 patients with HFrEF, administration of *S. boulardii* (1000 mg per day) for three months was associated with significant reductions in inflammatory markers [269]. However, in the GutHeart trial, supplementation with *S. boulardii* in patients with stable HFrEF did not result in any improvement in cardiac function in comparison with standard care [270].

In a rodent model of HF, administration of a probiotic formulation comprising *Lactobacillus* and *Bifidobacterium* genera resulted in a significant enhancement of cardiac functional parameters [271]. Other findings from animal studies suggest that supplementation with this probiotic promotes the production of SCFAs through modulation of the intestinal microbiome [272,273,274,275], and reduces the production of inflammatory cytokines in serum and colonic explants [276]. By reinforcing tight junctions and reducing intestinal permeability, *Lactobacillus* and *Bifidobacterium* help to preserve gut barrier function and minimize exposure to inflammatory triggers [277,278]. Despite these promising experimental findings, whether modulation of the gut microbiota through *Lactobacillus* and *Bifidobacterium* supplementation can improve outcomes in cardiometabolic HFpEF remains to be determined, highlighting the need for future mechanistic and clinical investigations.

In summary, probiotics such as *S. boulardii*, *Lactobacillus*, and *Bifidobacterium* show potential to modulate gut microbiota composition, enhance SCFA production, reduce systemic inflammation, and improve cardiac function in cardiometabolic HFpEF. However, probiotics alone may not exert optimal benefits, as SCFA generation requires fermentable substrates derived from dietary fibers. Thus, an effective therapeutic approach should combine probiotic supplementation with an adequate dietary fiber intake to synergistically restore gut–heart metabolic homeostasis. Evidence supporting the efficacy of this combination in cardiometabolic HFpEF is currently lacking, underscoring the need for future mechanistic and clinical studies.

## 6. Conclusions

Cardiometabolic HFpEF is increasingly recognized as a complex systemic disorder in which metabolic impairment and chronic low-grade systemic inflammation (i.e., metainflammation) converge on the heart. Evidence from both preclinical and clinical studies supports a role for the gut–heart axis in this process. Gut microbiota composition and functions may critically influence the systemic inflammatory-metabolic milieu that characterizes cardiometabolic HFpEF. Gut dysbiosis reduces SCFA-producing taxa, increases intestinal permeability, and promotes translocation of microbial products that may amplify inflammation and myocardial fibrosis. Conversely, restoration of SCFA homeostasis through dietary fibers, omega-3 rich diets, prebiotics, and selected probiotics may exert favorable effects on cardiac energy metabolism, cardiovascular functions, and immune regulation. These findings identify SCFAs as metabolically active mediators that bridge intestinal and cardiac health and as promising, low-risk nutritional targets for HFpEF management. However, despite these mechanistic insights mainly in experimental and preclinical models, clear and definitive evidence of clinical benefits from SCFA-targeted interventions in cardiometabolic HFpEF is still lacking. While experimental data strongly support mechanistic links between gut-derived metabolites and cardiometabolic pathways relevant to HFpEF, human evidence remains limited, heterogeneous, and largely associative. Given the limited variability of available human studies, small sample sizes, heterogeneous cohort characteristics, and potential for residual confounding, the role of the gut microbiota in HFpEF should be considered a promising but still preliminary area of research requiring further validation. Moreover, the generalizability of current findings is limited by the predominance of studies conducted in older, Western, and European populations. In addition, the applicability of SCFA-targeted nutritional strategies is limited by dietary adherence challenges, substantial interindividual variability in gut microbiota composition and responsiveness, and the current lack of robust HFpEF-specific randomized clinical trial evidence.

Accordingly, SCFA-targeted nutritional strategies should be viewed as adjunctive, hypothesis-generating approaches pending validation in well-designed, HFpEF-specific interventional trials. Some mechanisms—such as the impact of dietary fibers, prebiotics and probiotics on circulating SCFA levels, inflammatory markers, clinical and functional parameters in patient with HFpEF—are testable in near-term clinical trials, whereas others, including SCFA-mediated epigenetic modulation of inflammation, remain speculative and require further mechanistic research. Moreover, future research should aim to include more diverse cohorts to enhance external validity. Future studies should clarify causality, define optimal dietary strategies to enhance SCFA signaling and dose–response relationship, and validate microbiota-derived biomarkers for patient stratification. A precision-nutrition approach aimed at restoring gut microbial health and SCFA homeostasis may represent a promising strategy to modulate the gut–heart axis, potentially identifying it as a therapeutic target in cardiometabolic HFpEF.

## Figures and Tables

**Table 1:** Summary of the main mechanistic pathways through which SCFAs may influence cardiometabolic HFpEF, indicating proposed biological effects and type of supporting evidence. Human evidence is predominantly observational, while most mechanistic insights derive from experimental models.

Mechanistic Pathway	Proposed Biological Effects	Type of Evidence	Evidence
Cardiac metabolism	CPT1-independent mitochondrial uptake; more readily oxidized compared to ketones; support of metabolic flexibility under conditions of impaired fatty acid oxidation; alternative mitochondrial fuel in the failing heart	Animal; observational human (indirect metabolic associations)	[73,74]
Lipid metabolism and glucose homeostasis	Inhibition of lipolysis and promotion of lipogenesis; increase in hepatic cholesterol uptake from the bloodstream; promotion of adipocyte differentiation through upregulation of GPR43 and PPARγ; promotion of glucose uptake through activation of GPR41 and GPR43 and modulation of AMPK and PPARγ signaling; stimulation of insulin secretion through activation of GPR43; promotion of secretion of PYY and GLP-1	Animal; interventional human (non-HFpEF); observational human	[75,76,77,78,79,80,81,82,83,84,85,86,87,88,89,90,91,92]
Myocardial contractility	Improved post-ischemic myocardial contractility; attenuation of cardiac hypertrophy and fibrosis	Animal	[93,94,95]
Blood pressure regulation	Lower blood pressure; activation of GPR41 of smooth muscle and endothelial cells of low-resistance blood vessels promoting vasodilation and reduction of peripheral resistance; modulation of renal sodium handling	Animal; observational human	[94,96,97,98,99,100,101]
Heart rate and autonomic regulation	Enhanced vagal tone via stimulation of GPR41 and GPR43 resulting in reduced heart rate and hypotension	Animal	[95,102]
Inflammation and immunomodulation	Inhibition of NF-κB signaling through the activation of GPR109A; modulation of NLRP3 inflammasome through the binding with GPR43 and GPR109A; inhibition of the differentiation of pro-inflammatory Th1 and Th17 cells, and promotion of Treg cells differentiation; suppression of cyclooxygenase-2 and prostaglandin E2 expression	Animal; observational human	[103,104,105,106,107,108,109]
Epigenetic modulation	Inhibition of HDACs; regulation of gene transcription involved in inflammation, metabolism, and vascular function	Animal; *in vitro*	[109,110,111,112]

Abbreviations: AMPK: AMP-activated protein kinase; CPT1: carnitine palmitoyltransferase I; GLP-1: glucagon-like peptide 1; GPR109A: G-protein coupled receptor 109A; GPR43: G-protein coupled receptor 43; HDACs: histone deacetylases; NF-κB: Nuclear Factor kappa-light-chain-enhancer of activated B cells; NLRP3: NLR family pyrin domain-containing 3; PPARγ: proliferator-activated receptor γ; PYY: peptide YY; Th1 cells: T helper 1 cells; Th17 cells: T helper 17 cells; Treg cells: Regulatory T cells.

**Table 2:** Nutritional diets and compounds which modulate SCFA synthesis and production, exerting potential cardiometabolic benefits in cardiometabolic HFpEF.

Nutritional Intervention	Mechanisms of SCFA Modulation	Key Microbiota Changes	Cardiometabolic Effects	Evidence
High-fiber diets (e.g., Mediterranean diet, vegetarian diet, vegan diet)	↑ SCFA-producer spp. ↑ SCFA synthesis from fermentable fibers	↑ α-diversity ↑ *Lachnospira, Akkermansia, Bifidobacterium, Lactobacillus, Ruminococcus, Roseburia, Clostridium, Faecalibacterium, Dorea*	↓ CRP, LDL-C, total cholesterol ↓ HbA1c, ↑ GLP-1 secretion ↓ inflammation ↓ BP, left ventricular hypertrophy, and fibrosis in murine models	[115,228,229,230,231,232,233,234,235,236,237,238,239]
Omega-3-rich diets (DHA, EPA)	↑ SCFA-producer spp.	↑ *Eubacterium*, *Roseburia*, *Anaerostipes*, *Coprococcus Lachnospira*, *Lactobacillus*, *Bifidobacterium Blautia*, *Bacterioides*, *Coprococcus*	↓ inflammation in combination with dietary fibers intake	[240,241,242,243,244,245]
Prebiotics (RS, AXOS, ITF)	↑ SCFA synthesis	↑ *Bifidobacteria, F. prausnitzii, Ruminococcus, Anaerostipes*	↓ inflammation Improved myocardial fibrosis and hypertrophy in mouse models of DMC	[246,247,248,249,250,251,252,253,254,255,256,257,258,259,260,261,262,263,264]
Probiotics	↑ SCFA production when fermentable fibers are available	↑ *Lactobacillus* spp., *Bifidobacterium* spp., *S. boulardii*	↓ inflammation Improved gut barrier	[265,266,267,268,269,270,271,272,273,274,275,276,277,278]

Abbreviations: AXOS: arabinoxylan oligosaccharides; BP: blood pressure; CRP: c-reactive protein; DHA: docosahexaenoic acid; DMC: diabetic cardiomyopathy; EPA: eicosapentaenoic acid; GLP-1: glucagon-like peptide-1; HbA1c: hemoglobin A1c; ITF: inulin-type fructans; LDL-C: low-density lipoprotein cholesterol; RS: resistant starch; SCFA: short-chain fatty acids. ↑ and ↓ indicate an increase and a decrease, respectively.

## Data Availability

No new data were created or analyzed in this study.
